# Impact of Switching from Intermittently Scanned to Real-Time Continuous Glucose Monitoring Systems in a Type 1 Diabetes Patient French Cohort: An Observational Study of Clinical Practices

**DOI:** 10.1089/dia.2020.0515

**Published:** 2021-03-22

**Authors:** Yannis Préau, Martine Armand, Sébastien Galie, Pauline Schaepelynck, Denis Raccah

**Affiliations:** ^1^ APHM, University Hospital Sainte Marguerite, Department of Nutrition & Diabetes, Marseille, France.; ^2^ Aix Marseille Univ, CNRS, CRMBM, Marseille, France.

**Keywords:** Real-time continuous glucose monitoring, Intermittently scanned continuous glucose monitoring, Type 1 diabetes, Glycemic variability, Hypoglycemia, Insulin sensitivity

## Abstract

***Aim:*** Assess the impact of switching from intermittently scanned (FreeStyle Libre [FSL]) to real-time (Dexcom G4 platinum [DG4]) continuous glucose monitoring systems on glycemia control in type 1 diabetes (T1D) patients with high risk of hypoglycemia and/or elevated glycated hemoglobin (HbA1c).

***Methods:*** We conducted an observational study in 18 T1D adults with poor glycemic control on FSL. Ambulatory glucose profile data were collected during the last 3 months of FSL use before inclusion (M0 period), during the first 3 months (M3 period) and the last 3 months (M6 period) of DG4 use. Data were then expressed as 24-h averages. Biological HbA1c was measured for all three periods. Patients were their own-controls and statistics were performed using paired *t*-test or Wilcoxon for matched-pairs.

***Results:*** The switch to DG4 at M3 resulted in a higher time-in-range (TIR) 70–180 mg/dL (median [Q1;Q3], 53.1 [44.5;67.3] vs. 41.5 [28.5;62.0], *P* = 0.0008), and a lower time-below-range <70 mg/dL (TBR mean ± standard deviation (SD), 5.4 ± 3.7 vs. 10.9 ± 7.1, *P* = 0.0009) and in the glucose % coefficient of variation (%CV mean ± SD, 40.1 vs. 46.9, *P* = 0.0001). Mean (SD) changes were +10.3 (8.0) percentage points for TIR, −5.5 (5.8) percentage points for TBR, and −6.8 (5.8) percentage points for %CV. These results were confirmed at the M6 period.

***Conclusions:*** Switching from FSL to DG4 appears to provide a beneficial therapeutic option without changing insulin delivery systems, regardless of the origin of the patient's initial glycemic issue.

## Introduction

The main management goal for type 1 diabetes (T1D) is glycemic control.^1^ This is routinely assessed by the measurement of capillary blood glucose by self-monitoring (SMBG) practice providing a single “point-in-time” value, and of glycated hemoglobin (HbA1c), a reliable biomarker of chronic hyperglycemia events over the last 2–3 months, which is positively correlated with the risk of vascular complications.^2,3^ However, these measurements are limitative as not indicative of the intra- and interdaily glycemic excursions that lead to acute hypoglycemia and postprandial hyperglycemia of different amplitudes and durations, both linked to microvascular and macrovascular complications.^4,5^ This limitation can be resolved by using continuous glucose monitoring (CGM) systems, which enable a dynamic follow-up of interstitial glucose. This provides new data via electronic ambulatory glucose profile (AGP) report allowing a global vision of the patient's glucose profile, for example, glucose time-in-range 70–180 mg/dL (TIR), time in hypoglycemia or time-below-range <70 mg/dL (TBR), time in hyperglycemia or time-above-range >180 mg/dL (TAR), glucose variability such as glucose % coefficient of variation (%CV), mean interstitial glucose level, and glucose management indicator (GMI).^6^ To improve the management of diabetes, the ATTD international consensus now recommends specific thresholds to be achieved for AGP target parameters depending on age and vulnerability, taking care above all to limit time in hypoglycemia.^7,8^

To date, two types of CGM systems are available^9,10^: (i) intermittently scanned device (isCGM) or “flash monitoring,” implying scanning near the sensor, and devoid of alarms for hypo- or hyperglycemia excursions; (ii) real-time (rtCGM) device directly connected to the sensor, requiring a calibration by capillary blood glucose twice a day (except for the Dexcom G6), which can be set to alarms, warning the user of high or low glucose levels, and coupled to the insulin pump (sensor-augmented pump treatment). Due to the presence of threshold alarms, the rtCGM system would be more suitable for patients with hypoglycemia issue, while isCGM should suit to patients with elevated HbA1c. However, there are few comparative or switch studies available to confirm this hypothesis.^10–13^ While it needs to be explored, it is possible that T1D patients who use isCGM but continue to have a poor glycemic control despite using isCGM (severe hypoglycemia, chronic hyperglycemia) might benefit from rtCGM rather than modifying their insulin management while still preserving their comfort and autonomy.

In the present study, we thus aimed at exploring the impact of switching from isCGM to rtCGM systems on glycemic control in DT1 patients treated by continuous subcutaneous insulin infusion (CSII) or multiple daily insulin injections (MDI), having poor glycemic control either due to hypoglycemia issue or/and to elevated HbA1c.

## Materials and Methods

### Ethics

We performed a single-center observational study of clinical practices in T1D patients followed-up in the endocrinology and diabetology department at the University Hospital Sainte Marguerite/AP-HM of Marseille (France). The study was approved and registered by the AP-HM local Ethics Board (AP-HM Health Data Portal No. 2019-173) and conducted in accordance with the Declaration of Helsinki. All participants gave written informed consent for data sharing.

### Study genesis and objective

In our endocrinology and diabetology department, T1D patients switching from SMBG practice to isCGM device for at least 1 year can still exhibit a poor glycemic control, that is, having elevated HbA1c (>8%) and/or severe hypoglycemia episodes. Such patients, when treated with CSII, are therefore eligible for a predictive low-glucose suspend system, namely the MiniMed 640G with SmartGuard technology (Medtronic plc, Minneapolis, MN), which has been approved for reimbursement by the French health insurance since February 2018. For reasons of personal comfort, however, many patients prefer using their same insulin pump, primarily a patch pump (OmniPod; Insulet Corporation, Acton, MA). Thus, to improve glycemic control in these patients who are already treated by CSII or MDI without changing the insulin delivery systems, one alternative in our department is proposing a switch from isCGM (FreeStyle Libre [FSL]; Abbott Diabetes Care, Inc., Alameda, CA) to the rtCGM Dexcom G4 platinum (DG4; Dexcom, San Diego, CA), a device approved for reimbursement by the French health insurance system since June 2018.

The objective of this observational study was to assess the potential benefit of the switch from the FSL device to the DG4 one on glycemic control using parameters from the AGP report. The main evaluation criteria selected were time in glucose range 70–180 mg/dL (TIR), time in hypoglycemia <70 mg/dL (TBR), time in hyperglycemia >180 mg/dL (TAR), average interstitial glucose level, glycemic variability as %CV, GMI, the CGM system utilization rate, and biological HbA1c.

### Study design and patients

We have selected the electronic medical files of adult patients (≥18 years), FSL users for at least 1 year, who started to switch to DG4 between December 2018 to June 2019. The two reasons for changing device were either severe hypoglycemia events such as more than one episode of unconsciousness with a glucose level <0.54 mg/dL during the last 12 months with FSL and/or with hypoglycemia unawareness (Gold^14^ or Clarke^15^ score ≥4), either or/and elevated HbA1c level (≥8%).

The medical files were not retained if patients had used isCGM other than FSL, used regular paracetamol, experienced ketoacidosis during the last 3 months, were affected by a chronic progressive disease unbalancing diabetes (such as cancer, AIDS, viral hepatitis), were receiving corticosteroid therapy for any disorders, were pregnant or planning pregnancy or breast-feeding, or had severe visual or hearing impairment, or reduced manual dexterity.

**FIG. 1.**
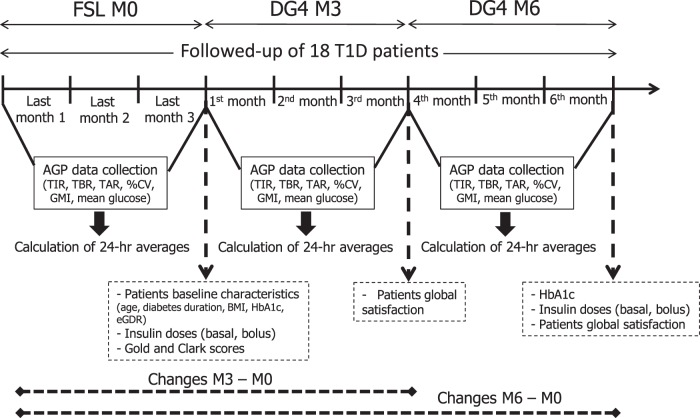
Study design. AGP, ambulatory glucose profile; BMI, body mass index; CV, glucose % coefficient of variation; DG4, Dexcom G4 platinum; eGDR, estimated glucose disposal rate; FSL, FreeStyle Libre; GMI, glucose management indicator; HbA1c, plasma glycated hemoglobin A1c; T1D, type 1 diabetes; TAR, time-above-range; TBR, time-below-range; TIR, time-in-target range.

Patients participated in a therapeutic patient educational (TPE) program conducted specifically in our clinical department by trained caregivers. This program allowed us to follow those T1D patients benefiting from the CGM switch over a period for 6 months through three different visits (Fig. 1):

Visit 1: initial group training session (five to six patients per session) (M0): recovery of AGP data for the last 3 months with FSL (i.e., 3 months before inclusion, considered as baseline), presentation of the DG4 device (e.g., handling of the sensor and monitor, calibrations, sensor's expected life time, AGP data download), individualized decision for hypo/hyperglycemia alarm thresholds (e.g., taking into account of the presence of hypoglycemic issue, habits of insulin adjustments during extraprandial hyperglycemia, hypoglycemia distress, and tolerance to repetitive audible alarms), collection of clinical and biological data (age, sex, date of birth, diabetes duration, body mass index (BMI), micro- and/or macrovascular complications, insulin doses, biological HbA1c, and severe hypoglycemia over the last 12 months), completion of Gold and Clarke questionnaires, and signature of informed consent for data sharing;
Visit 2: individual medical consultation at the third month (M3): collection of AGP data for the first 3 months with DG4, assessment of satisfaction with DG4, and collection of biological HbA1c value;
Visit 3: individual medical consultation at the sixth month (M6): collection of AGP data in the period comprised between 3 and 6 months of DG4 use, assessment of satisfaction with DG4, and collection of biological HbA1c value and of insulin doses.

### Data collection

Patient characteristics, that is, data on sex, age, diabetes duration, BMI, complications (retinopathy, nephropathy, coronary artery disease, carotid microangiopathy, and hypertension status), number of hypoglycemic episodes during the last year, and impaired hypoglycemia awareness were collected from the patient electronical files.

Data on biological HbA1c and insulin doses (basal, bolus) were collected retrospectively in the patient electronic files at 3 months before stopping the FSL device and at 3 months (for HbA1c only) and 6 months (HbA1c and insulin doses) after the switch to the DG4 device.

To better understand the metabolic profile of the patients, a validated score of insulin sensitivity status at baseline, that is, the estimated glucose disposal rate [eGDR mg/(kg·min)],^16^ was calculated using the following equation^17^: eGDR_BMI_ = 19.02 − (0.22 × BMI, kg/m^2^) − (3.26 × hypertension status [defined as 0 = no, 1 = yes]) − (0.61 × HbA1c, %), whereby the presence of hypertension was defined by the actual blood pressures (i.e., ≥140/90 mmHg) or current use of any antihypertensive agents. The higher the score the higher the sensitivity to insulin.

Data from the AGP report for the FSL and the DG4 systems were downloaded from the Freestyle Libre Libreview and Dexcom Clarity platforms, respectively. Both devices were used in compliance with manufacturers' licenses. Data were collected over 3 months at M0 (before inclusion during the last 3 months of FSL, i.e., at baseline), at M3 (over the first 3 months of DG4, i.e., during M0–M3 period), and at M6 (over the last 3 months of DG4, i.e., during M3–M6 period), and then expressed as 24-h averages. To note, the collection of data for 3 months, rather than the last month, as an example, was chosen for a best accuracy of the global vision of glycemic metrics in a real-life mode. Changes in glycemic control parameters were calculated by subtracting the averaged values obtained at M0 from the averaged values obtained at M3 (values at M3 − values at M0) or at M6 (values at M6 − values at M0).

### Statistical analysis

Since this was an explorative observational study, a power calculation was not possible. Data analysis was performed using GraphPad Prism version 8 (GraphPad Software, San Diego, CA). First descriptive statistics (mean, standard deviation (SD), median, and the first quartile Q1, i.e., the 25^th^ percentile, and the third quartile Q3, i.e., the 75^th^ percentile [Q1;Q3]) were performed and variable distributions were evaluated using the Shapiro–Wilk normality test. Data are thus presented as means ± SD when reaching normality or otherwise as medians [Q1;Q3]. When values followed a Gaussian distribution, the paired two-tailed *t*-test was applied, otherwise the nonparametric Wilcoxon signed-rank test (two-tailed) for matched-pairs was used, when comparing data from FSL to DG4 M3 or to DG4 M6, or from DG4 M3 to DG4 M6.

Association between patient characteristics at baseline (age, diabetes duration, HbA1c, BMI, insulin sensitivity status score, i.e., eGDR) and the changes of AGP parameters, or within glycemic control parameters, was tested using Spearman's rank correlation coefficient (or Spearman's rho denoted by “r”). *P*-value <0.05 was considered statistically significant.

## Results

Among the 25 analyzed patient files that met the defined selection criteria, 7 patients were not selected due to their voluntary termination of the DG4 device or their absence during a programmed TPE follow-up visit. Characteristics of the remaining 18 patients before the switch to DG4 are summarized in Table 1. Among them, 9 (50%) had elevated HbA1c (≥8%), 8 (44%) had experienced at least an episode of severe hypoglycemia in the previous year, 2 (11%) had both criteria, and 7 (39%) had a high insulin sensitive score [i.e., >8 mg/(kg·min)].^17^ Most patients were treated by CSII (*n* = 16; 89%). The average insulin doses calculated for the last 3 months were 0.26 ± 0.07 units/(kg·day) for basal dose and 0.54 ± 0.13 units/(kg·day) for total dose with FSL (at M0), and 0.27 ± 0.06 units/(kg·day) for basal dose and 0.57 ± 0.11 units/(kg·day) for total dose with DG4 (at M6).

**Table 1.** Characteristics of the Patients at Baseline

Number of T1D patients, *n*	18
Female, *n* (%)	12 (66.7)
Age, years (range)	48.3 ± 4.3 (31–75)
Diabetes duration, years (range)	29.3 ± 15.0 (7–60)
BMI, kg/m^2^ (range)	25.1 ± 4.9 (18–39)
Complications	
Retinopathy, *n* (%)	8 (44.4)
Nephropathy, *n* (%)	4 (22.2)
Coronary artery disease, *n* (%)	2 (11.1)
Carotid macroangiopathy, *n* (%)	3 (16.7)
Hypertension, *n* (%)	8 (44.4)
HbA1c, % (range)	8.07 ± 1.18 (6.3–10.7)
eGDR, mg/(kg·min) (range)	7.13 ± 2.20 (1.7–10.4)
Hypoglycemia risk	
Hypoglycemic episodes^a^ last 12 months, *n* (%)	8 (44.4)
Impaired hypoglycemia awareness,^b^*n* (%)	9 (50)

Data are mean ± standard deviation (range), unless stated otherwise.

^a^ Defined as ≥1 symptom of hypoglycemia with unconsciousness, blood glucose level <54 mg/dL.

^b^ On the basis of Clarke and/or Gold score ≥4.

BMI, body mass index; CSII, continuous subcutaneous insulin infusion; eGDR, estimated glucose disposal rate; HbA1c, plasma glycated hemoglobin A1c; T1D, type 1 diabetes.

During the last 3 months of using FSL, the mean ± SD average number of scans within 24 h was 6.2 ± 4.3. The means ± SD of average threshold hypoglycemic or hyperglycemic alarms were, respectively, 69 ± 4.0 or 240 ± 20.0 mg/dL when using DG4 device. Based on a 3-month average, the 24-h rate of sensor use was 68.8% ± 26.1% for FSL and 75.9% ± 20.8% or 74.6% ± 19.7% for DG4 at M3 and M6, respectively (no significant difference). Regarding individual data, 50%, 67%, and 56% patients showed a sensor use superior to 70% for FSL (range 78–100), for DG4 at M3 (range 71.5–97.9), and for DG4 at M6 (range 70.2–98.8), respectively. Concerning fingerstick calibration with DG4, the recommendation of capillary blood glucose monitoring every 12 h seems to have been respected by all patients, as we have verified on the various follow-up visits.

### Impact of switching from FSL to DG4 systems on glycemic control

The switch from FSL to DG4 at M3 led to a higher TIR, a lower TBR (<70 mg/dL), and a lower %CV of interstitial glucose (Table 2). Such results were confirmed with DG4 at M6. The TIR, TBR, and %CV were not different for DG4 use between M3 and M6. The TAR (>180 mg/dL), the average mean of interstitial glucose level, and both the biological HbA1c and GMI did not change between FSL and DG4.

**Table 2.** Impact of Switching FreeStyle Libre System to Dexcom G4 Platinum Over 3 and 6 Months on Glycemic Control Parameters

Parameters	FSL M0	DG4 M3	DG4 M6	*P* M0 vs. M3	*P* M0 vs. M6	Change*^a^*M3 – M0	Change*^a^*M6 – M0
HbA1c,^b^ %	8.07 ± 1.18	8.16 ± 1.02	8.19 ± 1.11	0.621	0.540	0.09 ± 0.75	0.12 ± 0.75
GMI,^c^ %	7.92 ± 1.50	7.58 ± 1.07	7.60 ± 1.23	0.143	0.139	−0.34 ± 0.95	−0.32 ± 0.88
TIR,^c,d^ % (70–180 mg/dL)	41.5 [28.5;62.0]	53.1 [44.5;67.3]	48.4 [41.5;69.2]	**0.0008**	**0.0015**	10.3 ± 8.0	9.5 ± 9.2
TBR,^c,d^ % (<70 mg/dL)	10.9 ± 7.1	5.4 ± 3.7	6.2 ± 5.2	**0.0009**	**0.0044**	−5.5 ± 5.8	−4.8 ± 6.1
TAR,^c,d^ % (>180 mg/dL)	44.7 ± 21.2	39.8 ± 18.3	40.0 ± 19.5	0.089	0.118	−4.9 ± 11.5	−4.7 ± 12.1
Average glucose,^c^ mg/dL	180.2 ± 42.9	171.2 ± 30.9	171.3 ± 35.4	0.178	0.152	−8.94 ± 27.0	−8.83 ± 25.0
CV,^c^ %	46.9 ± 8.1	40.1 ± 6.8	40.0 ± 7.6	**0.0001**	**0.0002**	−6.8 ± 5.8	−4.6 [−9.6;−3.7]

Data are mean ± standard deviation or median [Q1;Q3] from 18 T1D patients switching from FSL (M0) to DG4 followed-up for 3 months (M3) and for 6 months (M6). *P*-values in bold are statistically significant.

^a^ Change was calculated from DG4 parameters − FSL parameters, and is expressed as percentage points except for average glucose level (mg/dL).

^b^ Values after last 3 months of CGM used.

^c^ Data represent a 24-h average value calculated from the data collected on the ambulatory glucose profile report over 3 months (for M0 during the last 3 months of FSL use, for M3 during the first 3 months of DG4 use, and for M6 during the last 3 months of DG4 use).

^d^ Data are expressed as percent of time per day.

CGM, continuous glucose monitoring; CV, glucose % coefficient of variation; DG4, Dexcom G4 platinum; FSL, FreeStyle Libre; GMI, glucose management indicator; T1D, type 1 diabetes; TAR, time-above-range; TBR, time-below-range; TIR, time-in-target range.

**FIG. 2.**
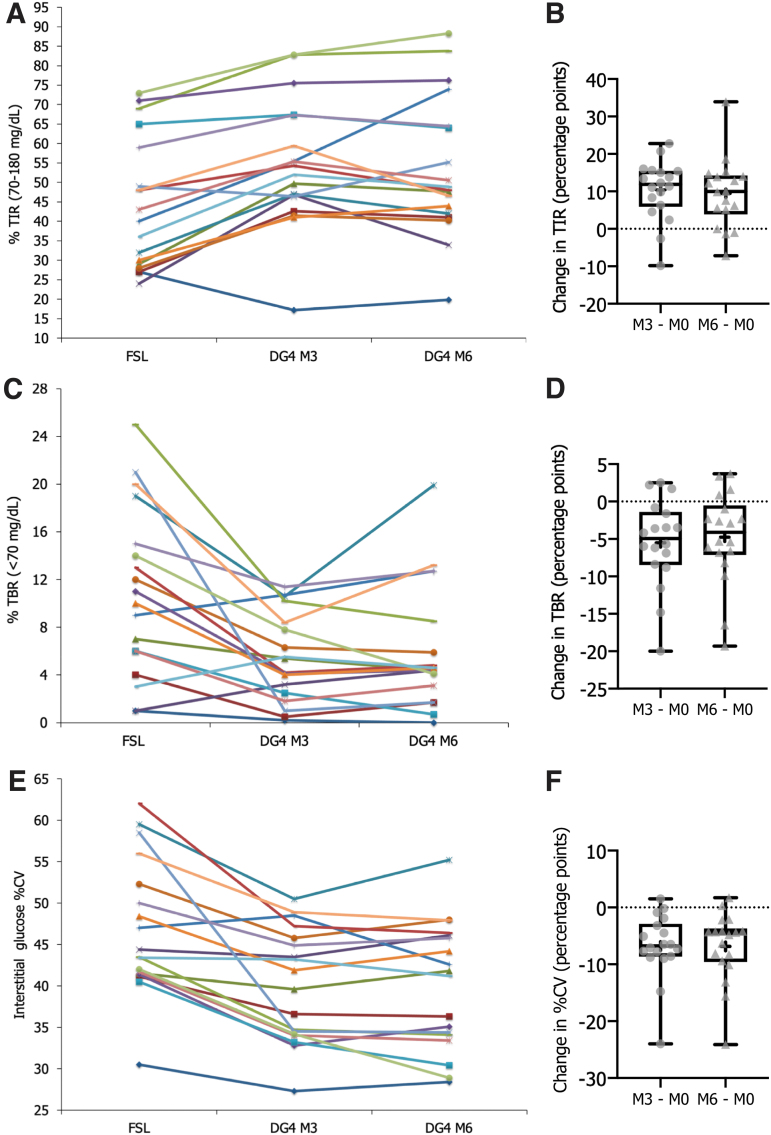
Individual evolution of TIR **(A)**, TBR <70 mg/dL **(C)**, and interstitial glucose %CV **(E)** with FSL and at the two periods with DG4, and box-and-whisker plot of changes in TIR **(B)**, TBR **(D)**, and %CV **(F)**. TIR, TBR, and %CV are expressed as percent of time per day and represent 24-h averages calculated from data collected over 3 months at the last 3-month period with FSL, at the first 3 months (M0-M3 period) and at the last 3 months (M3-M6 period) with DG4. Data represented as plots are median, first quartile (Q1 or 25^th^ percentile) and third quartile (Q3 or 75^th^ percentile), min and max, mean (as a cross), and all individual points for changes calculated from DG4 parameter at M3 or M6 − FSL parameters expressed as percentage points. Data are from 18 T1D followed-up patients. Color images are available online.

**FIG. 3.**
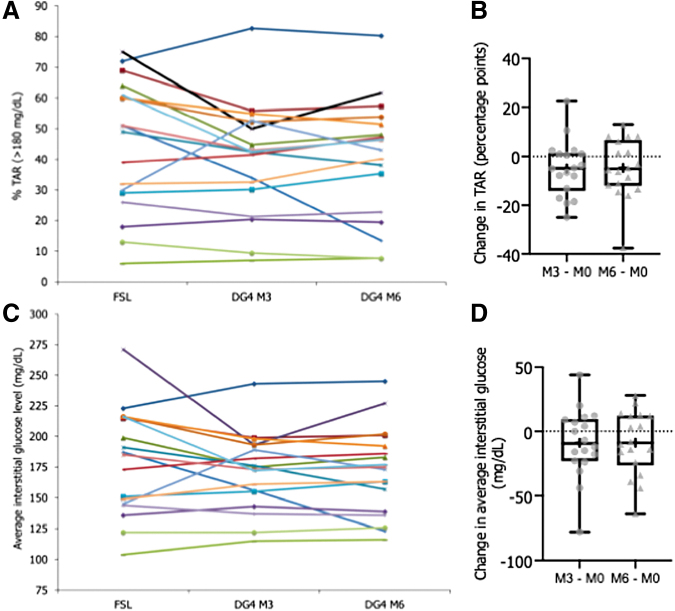
Individual evolution of TAR **(A)**, and average interstitial glucose level **(C)** with FSL and at the two periods with DG4, and box-and-whisker plot of changes in TAR **(B)** or average glucose **(D)**. TAR is expressed as percent of time per day and average glucose as mg/dL, and values represent 24-h averages calculated from data collected over 3 months at the last 3-month period with FSL, at the first 3 months (M0-M3 period) and at the last 3 months (M3-M6 period) with DG4. Data represented as plots are median, first quartile (Q1 or 25^th^ percentile) and third quartile (Q3 or 75^th^ percentile), min and max, mean (as a cross), and all individual points for changes calculated from DG4 parameter at M3 or M6 − FSL parameters expressed as percentage point for change in TAR or as mg/dL for glucose level. Data are from 18 T1D followed-up patients. Color images are available online.

Taking into consideration the individual patient data from FSL to DG4 at M3, only 2 patients out of 18 had no TIR increase (Fig. 2A) and 11 exhibited an increase equal or superior to the mean change value obtained (i.e., +11.0 to +22.8 percentage points, Fig. 2B), 15 patients decreased TBR (<70 mg/dL) (Fig. 2C) with 9 of them reaching a value equal or superior to the mean change value (−5.7 to −20 percentage points, Fig. 2D). There were 17 patients who improved their glucose variability (Fig. 2E) with 9 having a decrease equal or superior to the mean change value (−7.1 to −24.0 percentage points, Fig. 2F). In summary, there were 13 out of 18 patients showing improvements for these three glycemic targets. While the TAR (>180 mg/dL) mean did not significantly change, 11 out of 18 patients achieved a decrease (Fig. 3A) with 9 patients showing a value equal or superior to the mean value of the changes (−5.0 to −25.0 percentage points, Fig. 3B). Similarly, while the average level of interstitial glucose did not significantly change, 10 out of 18 patients achieved a decrease (Fig. 3C) with 9 patients showing a value equal or superior to the mean value of the changes (−12 to −78 mg/dL, Fig. 3D).

For individual responses between FSL and DG4 at M6, 4 patients out of 18 had no TIR increase (Fig. 2A) and 10 exhibited an increase equal or superior to the mean change value (+9.9 to +33.9 percentage points, Fig. 2B). For TBR (Fig. 2C), 14 patients showed a decrease with 9 of them reaching a value equal or superior to the mean change value (−5.3 to −19.3 percentage points, Fig. 2D). For glucose variability (Fig. 2E), 16 patients improved their %CV with 9 having a decrease equal or superior to the median change value (−4.8 to −24.1 percentage points, Fig. 2F). In the ending point, there were 9 out of 18 patients who showed improvements for these three glycemic targets. For TAR (Fig. 3A), 11 patients showed a decrease with 10 patients having a value equal or superior to the mean value of the change (−4.7 to −37.6 percentage points, Fig. 3B), and for the average interstitial glucose level (Fig. 3C), there were 10 patients who exhibited a decrease, of which 9 of them were equal or superior to the mean value of the change (−10 to −64 mg/dL, Fig. 3D).

To note, a reduction in biological HbA1c was obtained only in the T1D patients with initial elevated levels ranging from 8% to 10.7% (*n* = 9 patients) at 3 months and at 6 months (−0.3 to −1.2 percentage points for 6 out of the 9 patients).

Finally, neither severe hypoglycemia (event requiring the assistance from another person for administrating carbohydrates and/or glucagon, and/or brief hospitalization) nor diabetic ketoacidosis episode over the 6-month follow-up period was reported.

### Association between CGM metrics

**FIG. 4.**
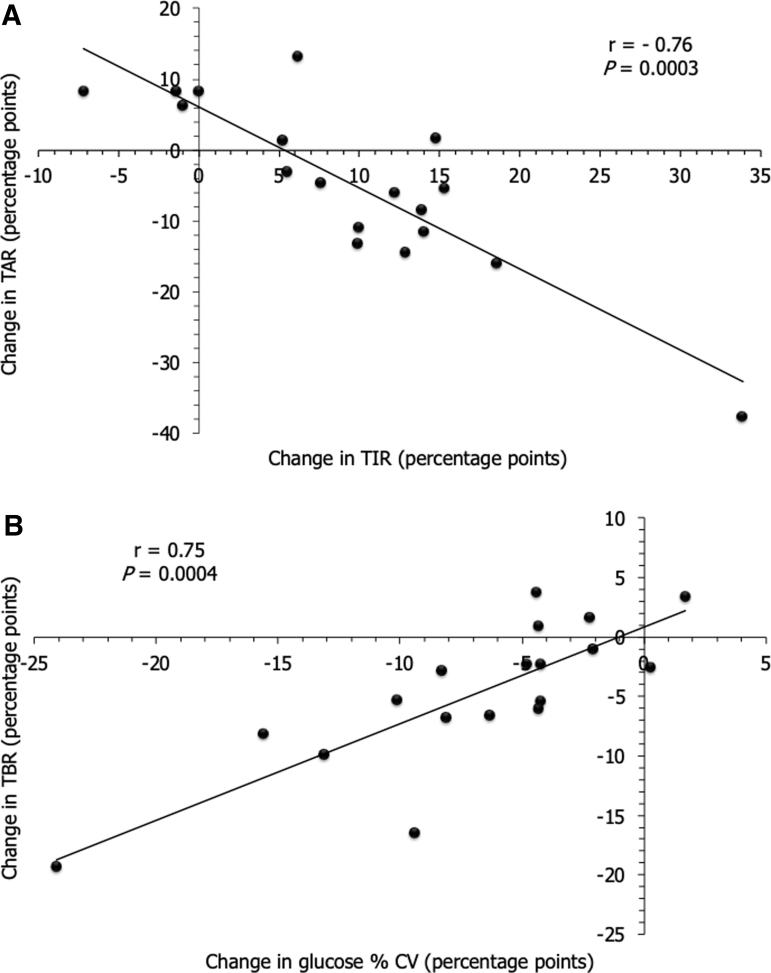
Linear association between changes obtained after switching from FSL to DG4 at M6 for TIR and TAR **(A)**, and glucose %CV and TBR **(B)**. Changes were calculated from DG4 6-month parameters − FSL last 3-month parameters (values at M6 − values at M0) in 18 T1D patients and are expressed as percentage points. Correlation analysis was performed using Spearman's rank correlation coefficient (or Spearman's rho) denoted by “r” on the figure (*P* < 0.05). Equation of the line for **(A)**: change in TAR = −1.1426 × [change in TIR] + 6.12. Equation of the line for **(B)**: change in TBR = 0.8093 × [change in %CV] + 0.80.

The change in TIR obtained after switching from FSL to DG4 at M6 was strongly inversely associated with the change in TAR (>180 mg/dL) (Fig. 4A; equation of the line: change in TAR = −1.1426 × [change in TIR] + 6.12), resulting in an inverse association with the change in GMI (Spearman's correlation coefficient *r* = −0.62, *P* = 0.006), but was not associated with the change in TBR (<70 mg/dL) (Spearman's correlation coefficient *r* = 0.15; *P* = 0.542). The change obtained in %CV was only strongly positively associated with the change in TBR (<70 mg/dL) (Fig. 4B; equation of the line: change in TBR = 0.8093 × [change in %CV] + 0.80).

### Association between patient characteristics and changes in metrics

No association was observed between baseline patient characteristics such as age, duration of diabetes, BMI, HbA1c, and changes in TIR, TBR, TAR, %CV, and average glucose level obtained after switching from FSL to DG4 at M6. Only eGDR, a score of insulin sensitivity status, showed a strong negative association with the change in TAR (Spearman's correlation coefficient *r* = −0.80; *P* < 0.0001) as well as a positive association with the change in TIR (Spearman's correlation coefficient *r* = 0.59; *P* = 0.009). We did not find any association between the average number of scans per day with FSL and the biological HbA1c value or the mean interstitial glucose level at M0.

## Discussion

We report herein a real-life observational study exploring in T1D the metabolic efficiency of switching from FSL to DG4 devices on a 6-month period in patients showing a poor glycemic control despite using the FSL system, that is, at high risk of hypoglycemia and/or with elevated HbA1c. The main clinical relevant outcome was a significant global improvement of three targets of the glycemic control during both 3 and 6 months of use of DG4, in accordance with the ATTD consensus recommendations,^7^ that is, an increase in TIR (70–180 mg/dL) (average changes +10.3 and +9.5 percentage points), less time in TBR (<70 mg/dL) (−5.5 and −4.8 percentage points), and a reduction in glucose variability attested by %CV (−6.8 and −6.9 percentage points). The benefits observed at the 3-month period were maintained over the entire 6-month study, and the absence of severe hypoglycemia corroborated well with the significant improvement on TBR. Importantly, such improvements occurred independently of the insulin delivery system since each patient kept the same patch pump or MDI therapy previously used with FSL when switching to DG4. In addition, the benefits for each patient participating in the study could indicate another perspective of the data that ought to be considered.

The unique published study comparable to our study design is the I HART CGM study extension phase conducted for 2 months in T1D adults on MDI with impaired hypoglycemia awareness but with HbA1c level lower than 8%, that investigated a switch from isCGM (FSL) system to rtCGM (Dexcom G5; note: not available in France).^11^ The magnitude of change we obtained for TIR (means: +10.3 or +9.5 percentage points at 3 or 6 months, ranges −2.6 to +22.8 or −1 to +33.9, *P* = 0.0008 or *P* = 0.0015) was higher compared to the I HART study (median: +3.5 percentage points at 2 months, ranges −0.4 to +7.2, *P* = 0.02), while the amplitude of the change for the TBR (<70 mg/dL) was close.

In our study, positive and clinically relevant outcomes could be explained by the educational approach at the initiation of the DG4 device, the need for capillary blood glucose calibration every 12 h, the presence of audible alarms to warn any hypoglycemia and hyperglycemia episodes thus encouraging greater patient involvement on a daily basis, and the significant proportion of CSII users more reactive to insulin dose adjustments based on threshold alarms or glycemic trends.^13,18^

The improvement in TIR (70–180 mg/dL) with DG4 in our T1D patients seemed mostly driven by the decrease in TAR (>180 mg/dL), with no relationship with the decrease in TBR (<70 mg/dL), as suggested by the Spearman's correlation coefficients. This is in agreement with a recent work published on AGP metrics relationship using simulation methods.^19^ While this hypothesis needs to be explored, both points taken together might suggest that a change in TIR could be mostly related to postprandial hyperglycemia and/or the dawn phenomenon rather than posthypoglycemic hyperglycemia.^7,20^ We also noted an inverse linear association between the change in TIR and the change in GMI, confirming the idea that TIR is a reliable marker of chronic exposure to hyperglycemia, usable in current practice.^7,20^ Conversely, the strong positive linear association found between the change in interstitial glucose %CV and the change in TBR (<70 mg/dL) suggested that the reduction in %CV was mainly explained by the decrease in exposure to hypoglycemia over 6 months. This is in accordance with the %CV being the most descriptive AGP metric for hypoglycemia excursions.^8^ This confirms hypoglycemia's major role on glycemic variability, an association to be considered in the context of the current debate on the link between these two glycemic disorders with the risk of vascular complications.^21,22^

Despite an inverse relationship between TIR and HbA1c described in the literature (which we observed herein for both CGM devices, data not shown), the significant increase in TIR was not accompanied by an improvement in HbA1c in our study.^7,20^ This absence of global change in HbA1c was in accordance with the I HART CGM study,^11^ while our T1D patients exhibited higher HbA1c (average ≥8%, range 6.3%–10.7%) and therefore a decrease in HbA1c could have been expected.^23^ In fact, such a reduction was obtained in our study, but only in six out of nine patients with initial elevated HbA1c.

Beyond threshold alarms, the TPE program and the fact that our T1D patients were mainly on CSII, and considering the high variability of the benefits between patients, another explanation for the improvement of metrics over 6 months might be the patients baseline characteristics (age, duration of diabetes, BMI, biological HbA1c, and insulin sensitivity evaluated by eGDR).^16,17^ Among all those characteristics, only the score of insulin sensitivity showed a linear association with the change in TAR and TIR. Patients with higher insulin sensitivity were more susceptible to benefit from a decrease in TAR and an increase in TIR than patients with insulin resistance. These data must be confirmed by the gold standard method of insulin sensitivity, hyperinsulinemic euglycemic clamp, as part of a larger scale study.^24^

Regarding the ATTD consensus recommendations, the threshold is >70% of readings or >16 h 48 min time per day for TIR (70–180 mg/dL), <4% or <1 h for time in TBR (<70 mg/dL), and ≤36% for %CV, for most individuals with T1D.^7,8^ Herein, no average reached such thresholds, whatever the CGM device used as in other study.^11^ Interestingly, when considering patient individual data, after 6 months on DG4 device, 4 out of 18 patients achieved the threshold for TIR (vs. 2 out of 18 on FSL), 6 exhibited <4% TBR (<70 mg/dL) (vs. 3 on 18 on FSL), and 7 reached threshold for %CV, that is, exhibited more stable glucose levels, while only one patient had a %CV below 36% on FSL. The two latter glycemic outcomes are clearly highly encouraging for a diabetologist even if the benefit with FSL could have been higher if the patients had performed more scans per day (mean 6.2 times/day), knowing that a metabolic benefit is reported for a minimum scan number of 15 times/day.^10^

Our observational study showed some limitations such as the small number of patients and the heterogeneity of the population (sex, age, diabetes duration, severe hypoglycemic episodes in only half of the patients, heterogeneous hypoglycemia awareness as Gold or Clarke score were not always ≥4, different HbA1c levels, and insulin sensitivity status). In addition, as for any study evaluating different CGM devices, it is advisable to remain cautious in the extrapolation of the results because the accuracy of different CGM devices (attested by mean absolute relative difference [MARD]) is not the same, in euglycemic range and even more in hypoglycemic range (e.g., calibration modalities, analysis software, sensitivity and specificity of glucose oxidase on the electrode, and behavior factors).^25^ More specifically for FSL, it is reported that the location of the sensor can influence the MARD (all patients of our study wore the sensor on the upper arm), and that the accuracy of the sensor could be slightly lower over the periods day 0 to 1 and day 13 to 14.^26,27^ We must also consider several data reporting a very high MARD for FSL (close to 30%) in the hypoglycemic range, which may contradict the possibility of comparisons with DG4 on TBR data.^28^ However, a recent comparative study between DG4 and FSL shows no significant difference in MARD for hypoglycemic range, which is rather reassuring.^29^

The strengths were that the study was conducted in real-life, in agreement with the French health insurance criteria, and on a longer period of time, that is, over a 6-month follow-up, with data collected for 3 months for each studied periods for a more precise global vision of the glycemic control of patients in real-life condition, with patients being her/his own control making the glycemic outcomes directly comparable with a clear conclusion as to the benefits for each patient.

## Conclusions

Our observational explorative study showed that switching from an isCGM system (FSL) to a rtCGM (DG4), without changing insulin delivery systems, was beneficial for improving several targets in glycemia control (i.e., TIR, TBR, and %CV). This was especially beneficial during the 6-month follow-up for 50% of our T1D patients who had continuing poor diabetes control with isCGM (i.e., at high risk of hypoglycemia or with elevated HbA1c). An exploratory study on a larger number of patients and for a longer period is necessary to confirm such encouraging results. In addition, it would be of interest to collect data about the device satisfaction, quality of life, and hypoglycemia fear and awareness evolution during follow-up through validated standardized questionnaires. This would provide guidance to the clinician in selecting the most appropriate system for each T1D patient in a personalized medicine setting.
